# Seed priming with polyethylene glycol regulating the physiological and molecular mechanism in rice (*Oryza sativa* L.) under nano-ZnO stress

**DOI:** 10.1038/srep14278

**Published:** 2015-09-30

**Authors:** Sheteiwy Mohamed Salah, Guan Yajing, Cao Dongdong, Li Jie, Nawaz Aamir, Hu Qijuan, Hu Weimin, Ning Mingyu, Hu Jin

**Affiliations:** 1 Seed Science Center, College of Agriculture and Biotechnology, Zhejiang University, Hangzhou 310058, China; 2 Department of Agronomy, Faculty of Agriculture, Mansoura University, Mansoura 35516, Egypt; 3 Zhejiang Nongke Seed Industry Limited Company, Hangzhou, 310021, China; 4 Faculty of Agricultural Sciences and Technology, Bahauddin Zakariya University Multan, 60000 Pakistan; 5 National Agricultural Technology Extension Service Center, China

## Abstract

The present study was designed to highlight the impact of seed priming with polyethylene glycol on physiological and molecular mechanism of two cultivars of *Oryza sativa* L. under different levels of zinc oxide nanorods (0, 250, 500 and 750 mg L^−1^). Plant growth parameters were significantly increased in seed priming with 30% PEG under nano-ZnO stress in both cultivars. Whereas, this increase was more prominent in cultivar Qian You No. 1 as compared to cultivar Zhu Liang You 06. Significant increase in photosynthetic pigment with PEG priming under stress. Antioxidant enzymes activities of superoxide dismutase (SOD), peroxidase (POD) and catalase (CAT) as well as malondialdehyde (MDA) contents were significantly reduced with PEG priming under nano-ZnO stress. Gene expression analysis also suggested that expression of *APXa*, *APXb*, *CATa*, *CATb*, *CATc*, *SOD1*, *SOD2* and *SOD3* genes were down regulated with PEG priming as compared to non-primed seeds under stress. The ultrastructural analysis showed that leaf mesophyll and root cells were significantly damaged under nano-ZnO stress in both cultivars but the damage was prominent in Zhu Liang You 06. However, seed priming with PEG significantly alleviate the toxic effects of nano-ZnO stress and improved the cell structures of leaf and roots in both cultivars.

Plants exposed to high concentrations of heavy metals experience changes in physiological, biochemical and molecular mechanisms of plant cells^1^. Uptake of nanoparticles (NPs) through primary roots is usually barred due to presence of suberinized exo- and endodermis. However, lateral root junctions are the primary sites through which NPs could enter the xylem via cortex and the central cylinder^2^. The higher concentrations of titanium dioxide (TiO_2_) enhanced the alterations in mitotic activity and chromosomal aberrations, indicating genotoxic effects of nanoparticles (NPs)^3^. Nano-ZnO stress shows more detrimental effects on germination and root growth of rice as compared to TiO_2_ nanoparticles^4^. Recently, Ng *et al.* studied the molecular downstream effects of ZnO nanoparticles on p53 signaling pathways, suggesting that ZnO nanoparticles might be sufficiently genotoxic to stimulate the DNA damage machinery and it might have caused DNA lesions because p53 was upregulated and phosphorylated with a concomitant decrease in cell cycle progression after seven days^5^. Furthermore, Giovanni *et al.* found that noncytotoxic zinc oxide NPs level (10 mg/L) could elevate the intracellular oxidative stress^6^. It has been observed that higher concentrations (2000 mg/L) of nano-Zn (35 nm) and ZnO (20 nm) inhibit the germination in ryegrass and corn, respectively^7^. Moreover, root length of the studied species was also inhibited with use of 200 mg/L nano-Zn and ZnO. In addition, phytotoxicity of nano-Al and Al_2_O_3_ significantly affect the root elongation of ryegrass and corn, and nano-Al enhance the root growth of radish and rape^8^. The physiology and biochemistry of the toxic effects of Zn in plants were likely to be similar to those reported for other heavy metals. In this regard, Chia *et al.* reported that ZnO NMs exert their toxic effects intracellularly^9^. However, Zn is not considered to be highly phytotoxic^10^.

Seed priming could be defined as a technique that controls the hydration level within seeds induce metabolic activities for germination but radical emergence is prevented. Previous studies have revealed that rice seed priming can enhance seed germination, vigour index and germination energy^11^. Polyethylene glycol (PEG) has been used frequently in plant water deficit studies to induce dehydration by decreasing water potential^12^. It is observed that priming with PEG can shorten the time to seed emergence and increases the germination percentage^13^, and improves salt tolerance^14^. Moreover, seed priming with PEG enhances the chilling tolerance^15^.

Zinc uptake in plants is critical as it plays many essential unique biological functions. The vast array of proteins use zinc for stabilizing their structures because it possesses in a functional form^16^. Zinc has major contribution to perform biochemical and physiological processes, even slight deficiencies may affect growth and yield. These all evidences inspired us to understand the molecular mechanisms of nano-ZnO uptake, translocation and storing of zinc in rice plants. This study provides new information on nanotoxicology, as we investigated the effects of seed priming with PEG on seed vigor, antioxidant enzyme activities and their gene expression of two rice cultivars under nano-ZnO stress. Moreover, the induction of antioxidative defense was also investigated at enzymatic as well as transcriptional level in order to disclose the toxicity mechanisms of ZnO in rice plants. This approach may enhance our understandings about the toxicity of engineered nanoparticles (ENPs) on this plant species. Thereafter, it could be helpful to improve the plant growth in ZnO polluted soils.

## Results

### Seed vigour and plant growth

The present study showed that germination percentage, germination index and energy of germination were significantly reduced at higher level of ZnO (750 mg L^−1^) in both cultivars over their respective controls (Table 1). The results also revealed that there was a significant difference in mean values of germination percentage, germination index and energy of germination which showed decreasing trend with an increase in ZnO concentrations and this decline was more obvious in cultivar Zhu Liang You 06 as compared with cultivar Qian You No. 1. As far as PEG priming was concerned, the result showed that seed priming with PEG (30%) resulted in a significant resistance to nano-ZnO stress (Table 1). Seed priming has been shown promising for hastening seed germination under stress as compared to the control in both cultivars. Likewise, leaf surface area was also statistically reduced under higher nano-ZnO concentration (750 mg L^−1^) as compared to the non-stressed plants. While, the lowest leaf surface area was observed under control conditions in both cultivars. However, the reduction in leaf area was more accentuate in cultivar Zhu Liang You 06 as compared to cultivar Qian You No. 1. In contrast, the maximum mean germination time were observed under higher nano ZnO stress (750 mg L^−1^) as compared to non-stressed plants in each cultivar. The data revealed that seed priming with PEG resulted in reduced mean germination time and significantly increased the leaf surface area in both cultivars irrespective of nano-ZnO concentrations (Table 1).

Exposure to different concentrations of nano-ZnO resulted in phenotypic changes as visualized by reductions in shoot and root lengths (Supplementary Fig. 1). As compared to control plants, significant gradual decrease in shoot and root length as well as seedling fresh and dry weight were observed in stressed plants with increasing nano-ZnO concentrations (Table 2). It was noticed that there was significant difference in shoot and root length as well as seedling fresh and dry weight at all nano-ZnO concentrations among the both cultivars. Nevertheless, the reduction in leaf area was more obvious in cultivar Zhu Liang You 06 as compared to cultivar Qian You No. 1. In addition, seed priming with PEG (30%) significantly increased the shoot and root length as well as seedling fresh and dry biomass irrespective of nano-ZnO concentrations. Similar trend was observed in case of seedling vigor index (Table 2).

### Photosynthetic pigments

**Figure 1:**
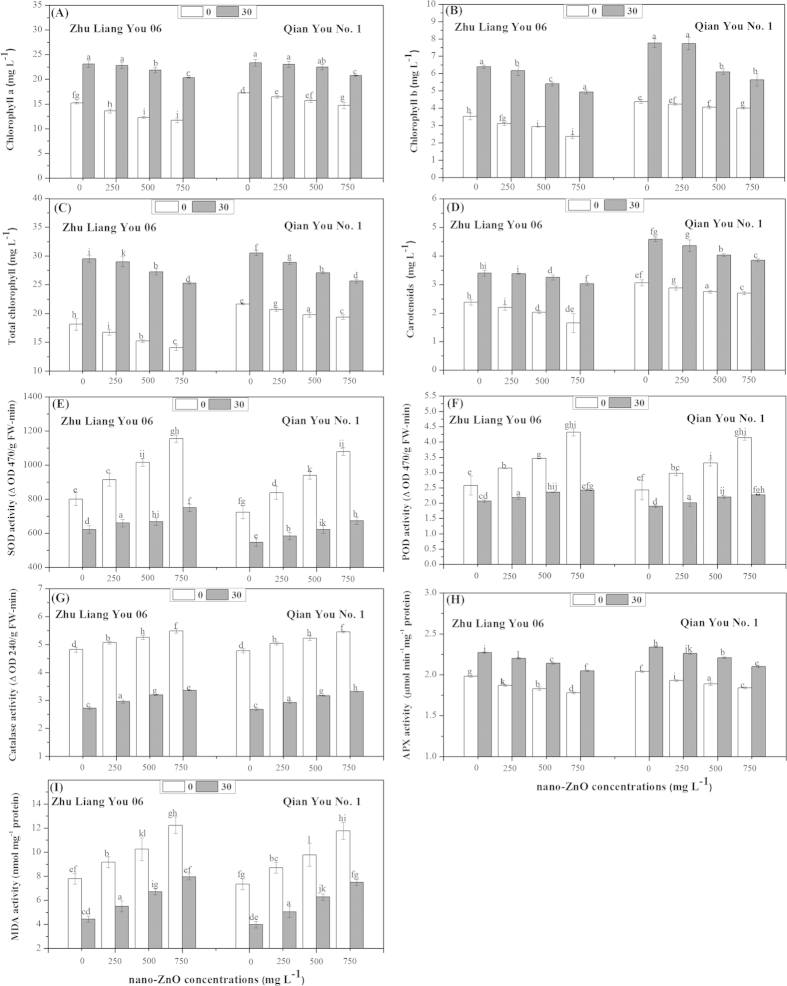
Effects of seed priming with PEG (30%) on (A) chlorophyll a, (B) chlorophyll b, (C) total chlorophyll, (D) carotenoids, (E) superoxide dismutase (SOD), (F) peroxidase (POD), (G) catalase (CAT), (H) ascorbate peroxidase (APX) and (I) malondialdehyde (MDA) contents in leaves of two cultivars of *Oryza sativa* under different concentrations of nano-ZnO stress.

Results showed that nano-ZnO stress alone significantly decreased Chl a, Chl b, total Chl, and carotenoid contents as compared to control (Fig. 1). However, the reduction in Chl a, Chl b, total Chl, and carotenoid contents was more clear upon exposure to 750 mg L^−1^ of nano-ZnO as compared to non-stressed plants. There was significant difference between the two cultivars in chlorophyll attributes under nano-ZnO concentrations. Results demonstrated that a linear decrease was observed in chlorophyll a, chlorophyll b, total chlorophyll and carotenoids contents under different nano-ZnO concentrations in two rice cultivars, this decrease was more pronounced in cultivar Zhu Liang You 06 as compared to cultivar Qian You No. 1 (Fig. 1). Seed priming with PEG significantly increased chlorophyll a (Chl a), chlorophyll b (Chl b), total chlorophyll (total Chl), and carotenoid contents under nano-ZnO stress in leaves of both rice cultivars as compared to control (Fig. 1).

### Antioxidants enzyme activities and malondialdehyde contents

Under nano-ZnO stress alone, activities of antioxidant enzymes and malondialdehyde contents were differentially modulated. The present study showed that higher concentration of nano-ZnO (750 mg L^−1^) significantly increased the activities of SOD, POD and CAT as well as enhanced the MDA contents in both cultivars as compared to non-stressed plants (Fig. 1). Interestingly, the increasing of SOD, POD, CAT activity and MDA contents was more noticeable in cultivar Zhu Liang You 06 as compared to cultivar Qian You No. 1. Taken together, priming seed with PEG (30%) resulted in a significant reduction in the levels of SOD, POD, CAT activities and MDA contents at 5% probability level as compared with control plants (Fig. 1E–G,I, respectively). In contrast, the data showed that higher concentration of nano-ZnO (750 mg L^−1^) decreased APX activity significantly in both cultivars as compared to those of non-stressed plants (Fig. 1H). However, the results showed that seed priming with PEG (30%) induced a significant increase in APX activity under different concentrations of nano-ZnO as compared to unprimed seeds and this increase in APX activity was more accentuate in cultivar Qian You No. 1 as compared to cultivar Zhu Liang You 06 irrespective of nano-ZnO concentrations (Fig. 1H).

### Gene expression

**Figure 2:**
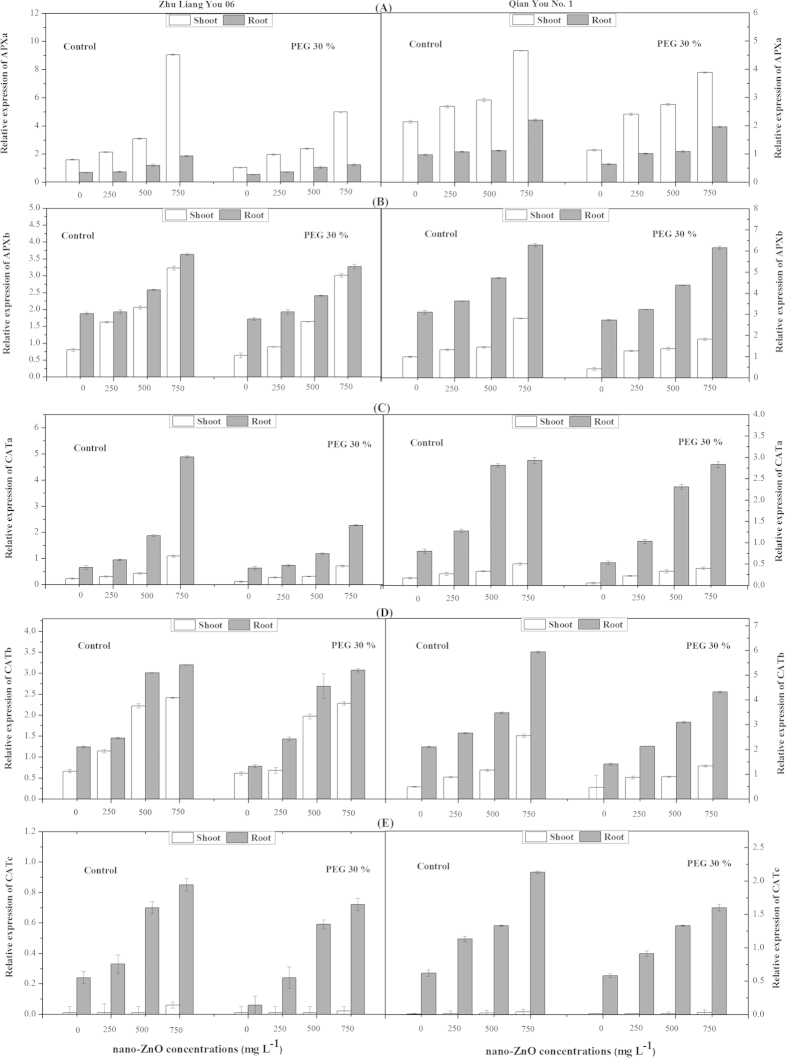
Effects of seed priming with PEG (30%) on gene expressions of (A) *APXa*, (B) *APXb*, (C) *CATa*, (D) *CATb* and (E) *CATc* in shoots and roots of two cultivars of *Oryza sativa* under different concentrations of nano-ZnO stress.

There was a significant difference in *APXa* expression in both root and shoot under different nano-ZnO concentrations. Significant up-regulation of *APXa* was observed in shoots after exposure to 500 and 750 mg L^−1^ nano-ZnO in both cultivars and the highest transcript levels of *APXa* was observed upon exposure to 750 mg L^−1^ as compared to non-stressed plants (Fig. 2A). Significant up-regulation of *APXa* was found in roots upon exposure to 500 and 750 mg L^−1^ nano-ZnO in both cultivars and this increase in transcript of *APXa* was more pronounced in cultivar Qian You No. 1 as compared to cultivar Zhu Liang You 06 (*p* < *0.01*) (Fig. 2A). The results indicated that seed priming with PEG (30%) induced significant reduction in transcript levels of *APXa* gene in both cultivars as compared with control (Fig. 2A). Similarly, exposure to different nano-ZnO concentrations induced a significant difference in *APXb* gene expression in both shoot and root of two cultivars. It was noticed that higher nano-ZnO concentration resulted in significant up-regulation in root and shoot of both cultivars (Fig. 2B). Irrespective of nano-ZnO concentrations, priming with PEG (30%) induced significant decrease in *APXb* gene expression in root and shoot of both cultivars as compared to the control (Fig. 2B).

Taken together, the data demonstrated that the highest concentrations of nano-ZnO (750 mg L^−1^) enhanced the expression levels of *CATa*, *CATb* and *CATc* genes in root and shoot of both cultivars as compared to non-stressed conditions (Fig. 2). However, the increase in transcript levels of *CAT* genes was highly significant in root as compared to shoot in both cultivars. It was observed that the reduction was more prominent in cultivar Qian You No. 1 as compared to cultivar Zhu Liang You 06. Irrespective of nano-ZnO concentrations, seed priming with PEG (30%) induced significant reduction in transcription levels of *CATa*, *CATb* and *CATc* genes in root and shoot of both cultivars as compared to control (Fig. 2C–E).

**Figure 3:**
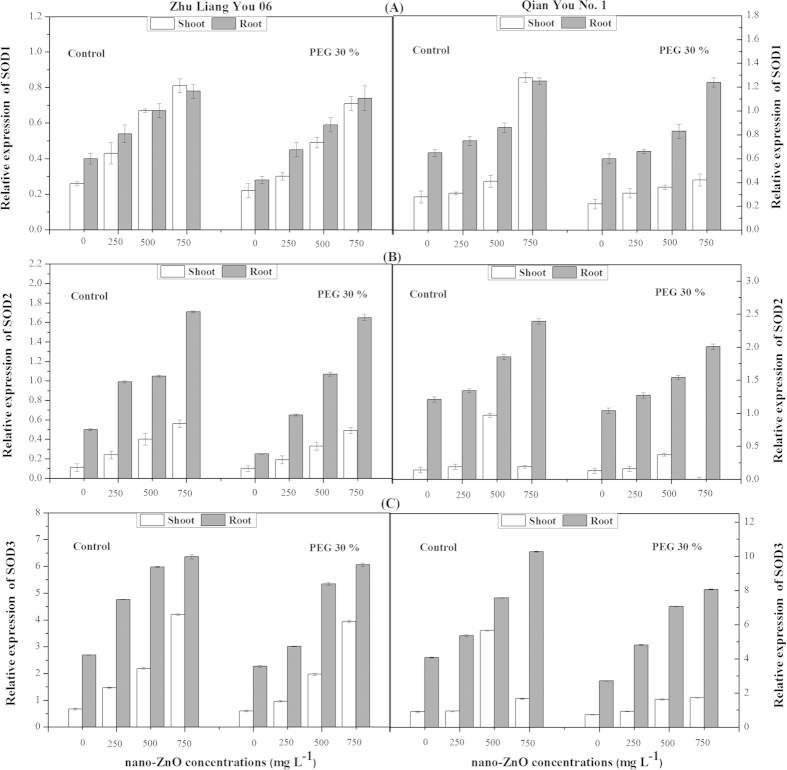
Effects of seed priming with PEG (30%) on gene expressions of (A) *SOD1*, (B) *SOD2* and (C) *SOD3* in shoots and roots of two cultivars of *Oryza sativa* under different concentrations of nano-ZnO stress.

Significant up-regulation in *SOD1* gene expression in root and shoot at all concentrations of nano-ZnO was observed (Fig. 3A). The highest expression of *SOD1* gene was observed in shoot upon exposure to 750 mg L^−1^ in both cultivars as compared to non-stressed plants. However, seed priming with PEG significantly decreased the *SOD1* expression under nano-ZnO stress conditions in both cultivars. This reduction in transcript levels of *SOD1* gene was more clearly in root and shoot of cultivar Qian You No. 1 as compared to cultivar Zhu Liang You 06. Moreover, a significant increase in *SOD2* expression was observed with increasing nano-ZnO concentration in both cultivars. Furthermore, data showed that seed priming with PEG induced decrease in transcription level of *SOD2* in both rice cultivars as compared to control plants (Fig. 3B). Likewise, exposure to high stress of nano-ZnO induced significant up-regulation in *SOD3* gene expression in both cultivars as compared to non-stressed plants. However, the up-regulation was clearly in root as compared to shoot irrespective of nano-ZnO concentrations (Fig. 3C). Results clearly described that seed priming with PEG induced decrease in *SOD3* gene expression in both root and shoot of two cultivars irrespective of nano-ZnO concentration (Fig. 3C).

### Ultrastructural changes

**Figure 4:**
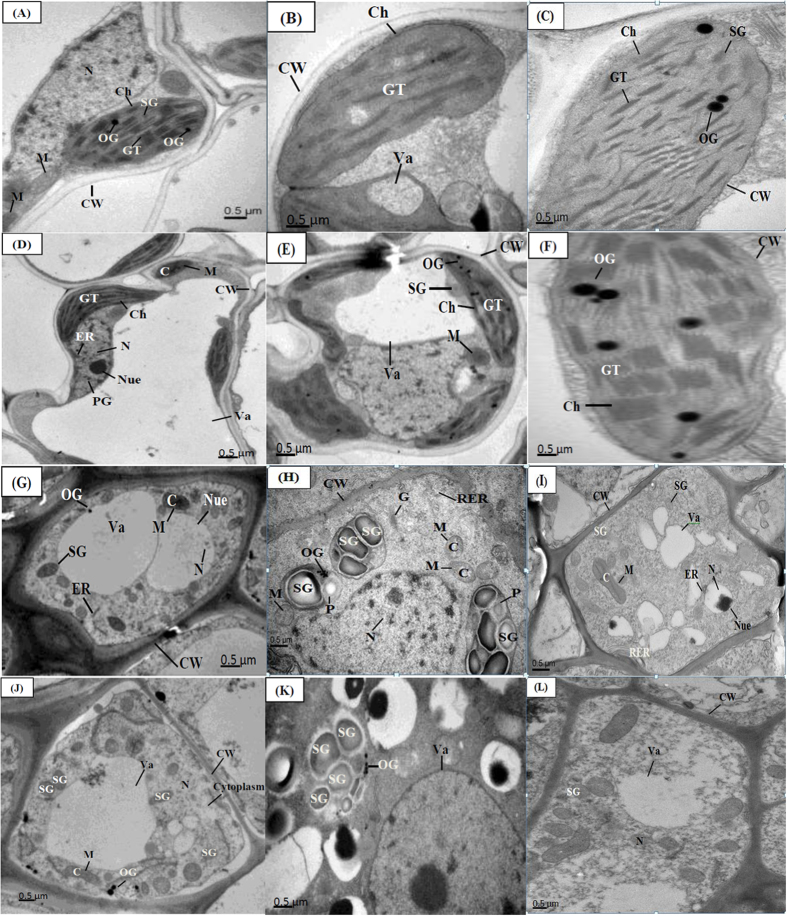
Electron micrographs of leaf mesophyll and root cell of two cultivars of *Oryza sativa* (cvs. Zhu Liang You 06 and Qian You No. 1) primed with PEG (30%) and grow under control and 750 mg L^−1^ of nano-ZnO concentration. (**A**) Leaf mesophyll cell of cultivar Zhu Liang You 06 at control level. (**B**) Leaf mesophyll cell of cultivar Zhu Liang You 06 primed with PEG (30%) and exposed to 750 mg L^−1^. (**C**) Leaf cell of cultivar Zhu Liang You 06 non-primed with PEG and exposed to 750 mg L^−1^. (**D**) Leaf mesophyll cell of cultivar Qian You No. 1 under control. (**E**) Leaf mesophyll cell of Qian You No. 1 primed with PEG (30%) and exposed to 750 mg L^−1^. (**F**) Leaf mesophyll cell of Qian You No. 1 non-primed with PEG (30%) and exposed to 750 mg L^−1^. (**G**) Root tip of cultivar Zhu Liang You 06 under control. (**H**) Root tip of cultivar Zhu Liang You 06 primed with PEG (30%) and exposed to 750 mg L^−1^. (**I**) Root tip of cultivar Zhu Liang You 06 non-primed with PEG (30%) and exposed to 750 mg L^−1^. (**J**) Root tip of cultivar Qian You No. 1 under control. (**K**) Root tip of cultivar Qian You No. 1 primed with PEG (30%) and exposed to 750 mg L^−1^. (**L**) Root tip of cultivar Qian You No. 1 non-primed with PEG and exposed to 750 mg L^−1^.

The ultrastructural changes in leaf mesophyll and root tip cells under control and higher nano-ZnO concentration (750 mg L^−1^) have been illustrated in Fig. 4. The TEM micrographs of leaf cell of cultivar Zhu Liang You 06 at control showed clean and thin cell walls, well-developed chloroplast with granule thylakoid, and the cell with rich contents and normal organelles (Fig. 4A). While, The TEM micrographs of leaf mesophyll of cultivar Zhu Liang You 06 (unprimed with PEG and exposed to 750 mg L^−1^) are displayed in Fig. 4C. It was found that this concentration destructed the cell wall and untidy arrangement of the thylakoid inside the chloroplast as compared to their respective control. Whereas, priming with PEG reduced the stress effect represented in clear cell wall (CW), well developed chloroplast (Ch) and tidy granule thylakoid inside the chloroplast (Fig. 4B), as well as no significant changes were found in the leaf mesophyll cell among two cultivars.

The TEM micrographs of root tip cells of cultivar Zhu Liang You 06 and cultivar Qian You No. 1 of control and priming treatments are demonstrated in Fig. 4(G–L). At control level, microscopic analysis showed that root tip cells of both cultivars presented clear cell walls, the cell had normal typical oval shaped mitochondria, a large size and well developed nucleus (Fig. 4G,J). While, The TEM micrographs of root tip of both cultivars primed with PEG (30%) and exposed to 750 mg L^−1^ showed that the rough ribosome (RER) was not swollen and had rich ribosomes (Fig. 4H,K). Moreover, the cell had more vacuoles and more deposition in the vacuole, and this reaction may reduce the effect of heavy metal on the cell (Fig. 4H,K). Whereas, the TEM micrographs of root tip of both unprimed cultivars and exposed to high stress (750 mg L^−1^) showed that the mitochondria and their cristae were swollen. The rough ribosome (RER) showed a little swollen and a part of the ribosomes was detached (Fig. 4I,L).

## Discussion

The present study was carried out to highlight the effects of seed priming with 30% PEG on the physiological and molecular mechanisms of two cultivars of *Oryza sativa* under nano-ZnO stress. In the present investigation, physiological parameters in terms of germination percentage, germination index, energy of germination, mean germination time and leaf surface area per plant significantly decreased with increasing nano-ZnO concentrations (Table 1). Recently, Lin and Xing investigated the effect of NPS in several physiological experiments and revealed that the phytotoxic dose of nanoparticles (NPs) varies between species under investigation. Furthermore, percent inhibitory concentrations (IC50) of nano-Zn and nano-ZnO were estimated to be near 50 mg L^−1^ for radish, and about 20 mg L^−1^ for rape and ryegrass^7^. The present study suggested that improvement of germination characters in primed seeds could be linked to physiological effects of pre-treatment. Indeed, primed seeds exhibited a faster imbibition as compared to unprimed seeds. In this trend, Nagarajan *et al.* also reported that improvement of germination by priming with PEG might be directly related to the modification of seed water relations^17^. Furthermore, this study therefore suggested that vacuums created inside the seed as a result of priming made water flow easier, thus contributing to tissue hydration. Additionally, Nagarajan *et al.* hypothesized that better performance of primed tomato seeds may be attributed to modifications of seed water binding properties during imbibition^17^. In this regard, Shaw and Hossain reported a significant inhibition in rice seed germination exposed to 0.5 mM nano-CuO treatment^18^. Nevertheless, Shaw *et al.* showed that nano-copper stress had no apparent effects on seeds germination of barley^19^.

The data further depicted a decreasing trend in seedling dry and fresh weight with increasing nano-ZnO concentrations (Table 2). According to the results, it was found that nano-ZnO stress caused significant toxic effects on the morphological characteristics of *Oryza sativa* as compared to non-stressed plants. A significant decrease in growth was found and this might be due to the adverse effects of nano-ZnO stress on the roots so the plants were not able to take up nutrients and continue their normal activity. Several study indicated that heavy metals stress induced a decrease in fresh and dry biomass which can inhibit the photosynthetic electron transport chain and ultimately result in biomass reduction^20^. Moreover, inhibition of plant biomass might be due to inhibition of photosynthesis, inactivity of both photosystem II and the enzymes of carbon reduction cycles^10^.

In present study, a significant reduction in chlorophyll attributes and carotenoids contents was observed in *Oryza sativa* seedlings exposed to nano-ZnO stress (Fig. 1A–D). The significant reduction in chlorophyll attributes, as observed in this study, might be due to the reduced biomass or as a result of excess lipid peroxidation of chloroplast membranes due to oxidative stress^21^. Similarly, significant reduction in chlorophyll attributes as a result of silver nanoparticles (AgNPs) exposure had also been reported from *Spirodela polyrhiza*^22^ and *Arabidopsis thaliana*^21^. Moreover, significant reduction in total carotenoids content upon exposure to AgNPs and may negatively affect the plants ability to quench excess ROS^23^.

Data regarding the antioxidant enzyme activities showed that SOD activity was increased in rice plants growing under toxic levels of nano-ZnO stress (Fig. 1E). Increase in SOD activity in response to stress appeared to be probably due to de-novo synthesis of the enzyme protein^24^. SOD activity has been reported to increase under salinity^25^ and heavy metals toxicity^26^. The present study indicated an enhancement in POD activity under nano-ZnO stress (Fig. 1F) which suggested that this enzyme served as an intrinsic defense tool to resist ZnO induced oxidative damage in rice plants. Under salinity and metal toxicity conditions, the level of POD had been used as potential biomarker to evaluate the intensity of stress^26^. Increased POD activity has been documented under a variety of stressful conditions such as water stress^27^, salinity^28^ and toxic levels of Al, Cu, Cd, Zn^26^. Moreover, increased POD activity in Pb-stressed seedlings might be due to increased release of peroxidases localized in the cell walls^29^.

Results showed that CAT activity was significantly enhanced under different nano-ZnO concentrations i.e. 500 and 750 mg L^−1^. While, APX activity decreased in relation to different nano-ZnO concentrations (Fig. 1G,H). The increase of CAT activity in leaves under nano-ZnO stress suggested that its effective scavenging mechanism to remove H_2_O_2_ resulted from metal stress caused oxidative damage^30^. However, reduction in antioxidant enzyme activities might be due to the oxidative stress, inhibition of enzyme synthesis and change in the assemblage of enzyme subunits^31^.

The MDA content is often measured as a suitable physiological index to reflect the degree of lipid peroxidation (MDA) and stress tolerance in plants^32^. Change in MDA concentration has been used as a parameter to assess oxidative stress damages to lipid membranes^33^. In the present study, MDA contents were enhanced in both cultivars under nano-ZnO stress. However, seed priming with PEG decreased MDA contents under different concentrations of nano-ZnO (Fig. 1I). These results are in consistent with the observations, showing that ALA could attenuate abiotic stress, probably due to its ability to act as antioxidant scavenging ROS^34^.

Gene expression in response to heavy metals stress is usually studied at the level of mRNA abundance because this gives a more precise estimate of antioxidant gene activation than enzyme activity. For this reason, we studied multiple components of the antioxidant enzymes at both the enzymatic and transcriptional level. Expression of *APX* genes had been demonstrated to be enhanced in plants by NaCl treatment^35^. In agreement with our results, Gupta *et al.* reported that *APX* transcripts might be up-regulated by increased levels of H_2_O_2_ in tobacco chloroplasts as results of Cu-Zn-superoxide dismutase overexpression^36^. Further, it was also found that catalase genes (*CATa, CATb* and *CATc*) were significantly enhanced in both root and shoot under different concentrations of nano-ZnO (Fig. 2C–E). Significant increase in catalase transcription, despite no significant change was observed in catalase enzymatic activity of leaves of *Arabidopsis thaliana*^37^. These discrepancies can be due to the presence of multiple allo- or isozymes. Alternatively, nano-ZnO stress could cause an enhanced break down of the proteins, which in turn also leads to an enhanced transcription. In the present study, it was observed that the expression levels of different *SOD* genes (*SOD1, SOD2* and *SOD3*) also showed significant induction upon exposure to different concentrations of nano-ZnO (Fig. 3A–C) showing that oxidative stress caused in different cellular compartments of the cell as a result of nano-ZnO exposure. In general, high significant changes in the transcript level of most antioxidative genes were found, but not all genes showed similar responses in roots and shoots. Most of genes were significantly up-regulated in root as compared to shoots that may be due to that plant roots are the first point of contact for these toxic of nano-ZnO. These observations supported the view of Smeets *et al.* who indicated that the underlying mechanism of oxidative stress was different in the roots and leaves. Additionally, the generation of superoxide and the lipoxygenase activity are the main causes of oxidative stress in the roots, whereas in the leaves H_2_O_2_ seemed to be an important candidate. Whether, this H_2_O_2_ was produced locally as a result of increased Cd content of the leaves, or whether it arrived as a signal from the roots, remains to be elucidated^37^.

In the present study, the ultrastructural changes occurred in different parts of plant cells were found to be dose-dependent. The leaf mesophyll cells were significantly damaged at 750 mg L^−1^ nano-ZnO in both cultivars, however the damage was prominent in cultivar Zhu Liang You 06 as compared to cultivar Qian You No. 1 (Fig. 4A–F). The number of osmiumphobilic granule and starch grains increased in leaf cells in both cultivars under nano-ZnO stress which indicated that plants might undergo in stress under metal stress^30^. The root tip cells of cultivar Zhu Liang You No. 1 were significantly damaged under nano-ZnO stress as compared to cultivar Qian You No. 1. The disappearance of different organelles and ruptured cell structure was found in cultivar Zhu Liang You 06 under nano-ZnO stress (Fig. 4L). Previous study showed that heavy metal toxicity damaged the root tip cells of *Brassica napus*^38^. Moreover, Daud *et al.* observed the presence of plasmolysis in root tips cells of cotton under the different concentrations of Cd^39^. However, seed priming with PEG significantly improved the cellular ultrastructure of leaf and root in both rice cultivars under nano-ZnO stress conditions (Fig. 4B,E,H,K). Cell structure was improved when citric acid applied with Cd-treated plants in *Juncus effusu*s^40^. Thus, it could be concluded that seed priming with PEG (30%) could decrease antioxidant enzymes activities, leading to avoid cell ultrastructure damage.

In summary, the exposure of rice seeds to nano-ZnO had a clear phytotoxic effect on the physiological, antioxidant enzymes and molecular mechanism of rice seedlings. Data suggested that seed priming with PEG (30%) alleviated the toxicity of nano-ZnO to *Oryza sativa* seeds and improved both germination and early seedling growth under different concentrations of nano-ZnO. Moreover, this study depicted that Qian You No. 1 proved to be more resistant to nano-ZnO stress as compared to Zhu Liang You 06 cultivar. The electron microscopic study revealed that ruptured cell in leaf and root tip were more prominent in Zhu Liang You 06 as compared to Qian You No. 1 cultivar under control and high stressed plants.

## Methods

### Engineered ZnO nanorods

**Figure 5:**
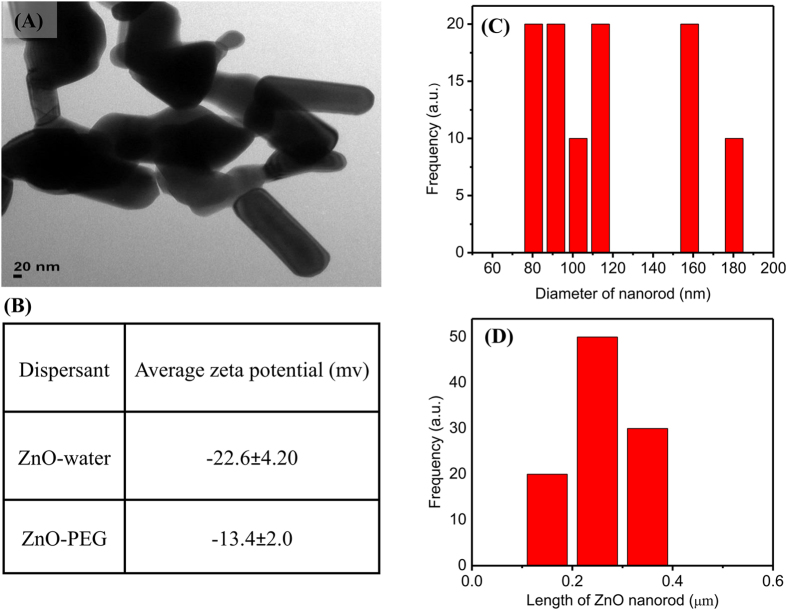
(**A**) Transmission electron microscopy (TEM) after dispersed in Milli-Q water of nano-ZnO scale 20 nm. (**B**) Average zeta potential of nano-ZnO in water and nano-ZnO in PEG. (**C**) Diameter of ZnO nanorod (nm). (**D**) Length of ZnO nanorod (μm).

Dispersions of nanorods used in this study were prepared at the laboratory of the Center of Nanotechnology, College of Agriculture and Biotechnology, Zhejiang University, China. Zinc oxide nanorods (nano-ZnO) were prepared from commercial ZnO nanopowder (Sigma-Aldrich, USA) by dispersing nanorods in Milli-Q water through ultrasonication (300 W, 40 kHz) for 30 min. The TEM Images of ZnO nanorods were taken by using scion image processing software (Fig. 5A). Average zeta potential of nano-ZnO in water and nano-ZnO in PEG was measured by Zetasizer nano (Zs3590, Malvern, United Kingdom) (Fig. 5B). The average length and diameter of the ZnO nanorods were 0.25 nm and 117 μm respectively, measured by Nano Measured software (Fig. 5C,D).

### Plant material and growth conditions

Seeds of two cultivars of *Oryza sativa*, L. (cvs. Zhu Liang You 06 and Qian You No. 1) were obtained from the Center of Seed Science, College of Agriculture and Biotechnology, Zhejiang University, China. The seeds were surface sterilized with 0.5% NaClO solution for 15 min and then washed several times with tap water followed by washing with sterilized distilled water for thrice to remove the traces of the disinfectant. The sterilized seeds were primed at 15 °C in darkness for 24 hr with PEG 30%. Then, seeds were dried back to their original moisture contents at room temperature. The unprimed dry seeds were used as control (CK). After priming, seed germination tests were carried out. Fifty seeds for each treatment were placed in a plastic germination box (12 cm × 18 cm) and each treatment replicated three times. Then, seeds were incubated in a germination chamber at 25 °C under alternating cycle of 8 hr illumination and 16 hr darkness for 14 days^41^. The incubated seeds were exposed to different concentrations of nano-ZnO (NPs ≤ 100 nm) i.e. 0, 250, 500 and 750 mgL^−1^. The nano-ZnO concentrations were based on findings from preliminary studies. Nano-ZnO at 250 mgL^−1^ concentration showed a little damage to plant growth. While, nano-ZnO at 750 mgL^−1^ concentration imposed a significant damage to plant growth. Whereas, concentrations higher than 750 mgL^−1^ were too toxic for plant growth. Furthermore, ZnO nanorods size was determined based on the pre-experiment study. It was clearly observed that ZnO nanorods larger than 100 nm showed a greater level of inhibition of seed germination and seedling growth. For this reason, only NPs ≤ 100 nm of ZnO were used.

### Physiological parameters

The germinated seeds were counted daily for 14 days. Then, the germination percentage was calculated at the 14^th^ Day. The germination index and mean germination time were calculated as the following equation^42^:


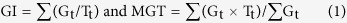


where G_t_ is the number of germinated seeds on Day t, T_t_ is time corresponding to G_t_ in days. Energy of germination was calculated as the percentage of germinating seeds at the 4^th^ day after sowing relative to the number of seeds tested^43^. For seedling parameter measurements, fourteen day old seedlings were harvested, the length of shoots and roots, and the leaf area of randomly selected ten plants per treatment were measured manually. Dry and fresh weight of the rice seedlings was determined separately. Fresh weight of ten seedlings per treatment were weighed immediately after harvesting and then placed into an oven at 80 °C for 24 hr. The dried samples were weighed immediately after removal from the oven until the weight became stable^44^. For RNA extraction, until their respective analysis. Seedling vigor the control and nano-ZnO exposed seedlings, frozen in liquid nitrogen and stored at −80 °C until their respective analysis. Seedling vigor index was calculated as the following formula^45^:


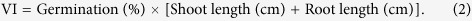


### Photosynthetic pigments determination

The amount of photosynthetic pigments in terms of chlorophyll a, chlorophyll b, total chlorophyll and carotenoids were determined according to the method of Hartmut *et al.* In brief, Fresh leaf tissues (0.2 g) was homogenized in 3 mL ethanol (95%, v/v). The homogenate was centrifuged at 5000 g for 10 min and the supernatant was extracted. Then, 9 mL ethanol (95%, v/v) was added to 1 mL aliquot of the supernatant. After that, the mixture was determined by monitoring the absorbance at the wavelengths 665, 649, and 470 nm by using spectrophotometer^46^. The following equations were used for the calculation of pigment amounts:


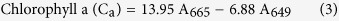



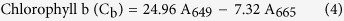



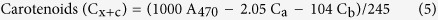



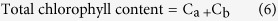


The amounts of pigments were calculated as milligrams per litre of plant extract.

### Antioxidant enzyme activities and malondialdehyde contents

In order to measure the antioxidant enzyme activities (SOD, CAT, APX and POD) and malondialdehyde (MDA) contents, leaf samples (0.5 g) were taken per treatment and homogenized in 8 mL of 50 mM potassium phosphate buffer (pH 7.8) under ice cold conditions. Homogenate was centrifuged at 10,000 g for 20 min at 4 °C and the supernatant was used for the determination of the following enzyme activities. Superoxide dismutase (SOD) activity was assayed by measuring its ability to inhibit the photochemical reduction of nitroblue tetrazo-lium (NBT)^47^. NBT reaction solution contained 50 mmol L^−1^ phosphate buffer (pH 7.8), 13 mmol L^−1^ methionine, 75 μmol L^−1^ NBT, 2 μmol L^−1^ riboflavin, 0.1 mmol L^−1^ EDTA. The reaction mixture was 3.1 mL, which contained 3 mL NBT reaction solution and 0.1 mL of enzyme extract. Reaction was started by adding 2 μmol L^−1^ riboflavin and placing the reaction tubes under 15 W fluorescent lamps for 15 min. A complete reaction mixture without enzyme extract served as a control. The photo reduction of NBT was measured at 560 nm and one unit of SOD was defined as being present in the volume of extract that caused inhibition of the photo- reduction of NBT by 50%. Guaiacol peroxidase (POD) activity was measured with guaiacol as the substrate in a total volume of 3 mL^48^. The 3 mL reaction mixture consisted of 2.7 mL phosphate buffer (25 mM, pH 7.0), 0.1 mL guaiacol (1.5%), 0.1 ml H_2_O_2_ (0.4%) and 0.1 mL of enzyme extract. Increase in the absorbance due to oxidation of guaiacol (E = 25.5 mM^−1^ cm^−1^) was measured at 470 nm. The enzyme activity was calculated in terms of l M of guaiacol oxidized g^−1^ FW min^−1^ at 25 ± 2 °C.

Catalase (CAT) activity was measured by reduction in absorbance at 240 nm due to the decline of extinction H_2_O_2_. The 3 mL reaction mixture containing 2.8 mL phosphate buffer (25 mM, pH 7.0), 0.1 mL H_2_O_2_ (0.4%) and 0.1 mL enzyme extract was used. The reaction was started with the addition of H_2_O_2_^49^. The enzyme activity was calculated in terms of l M of H_2_O_2_ g^−1^ FW min^−1^ at 25 ± 2 °C. Ascorbate peroxidase (APX) activity was measured according to^50^. The assay depended on the decrease in absorbance at 290 nm as ascorbate was oxidized. The 3 mL reaction mixture consisted of 2.7 mL phosphate buffer (25 mM, pH 7.0), 0.1 mL ascorbate (7.5 mM), 0.1 mL H_2_O_2_(0.4%) and 0.1 mL of enzyme extract. The reaction started by addition of H_2_O_2_. The enzyme activity was calculated in terms of (μmol min^−1^ mg^−1^ protein) at 25 ± 2 °C.

Malondialdehyde (MDA) concentration was determined as 2-thiobarbituric acid (TBA) reactive metabolites^20^. About 1.5 mL extract was homogenized in 2.5 mL of 5% TBA made in 5% trichloroacetic acid (TCA). The mixture was heated at 95 °C for 15 min, and then quickly cooled on ice. After centrifugation at 5,000 g for 10 min, the absorbance of the supernatant was measured at 532 nm. Correction of nonspecific turbidity was made by subtracting the absorbance value measured at 600 nm. The concentration of MDA was calculated in terms of (nmol mg^−1^ protein).

### Analysis of gene expression

Frozen leaf tissue (100 mg) was grinded thoroughly in liquid nitrogen using a pestle and mortar. Total RNA was isolated from the shoot and roots at Ck, 250, 500 and 750 mgL^−1^ of nano-ZnO stressed seedlings by using RNA isolation (Takara, Japan) following the manufacturer’s instructions. The concentration of the RNA was determined by NanoDrop 2000/2000c (Thermo Scientific, USA). The RNA purity was also checked spectrophotometrically by means of the 260/280 nm ratio. The primers for *APXa, APXb, CATa, CATb, CATc, SOD1, SOD2, SOD3* and *ACT1* genes were designed using online NCBI Primer-blast (http://www.ncbi.nlm.nih.gov/tools/primer-blast/index.cgi?link_LOC=BlastHome). cDNA was synthesized using Primer Script RT reagent Kit (Takara, Japan) from 1 μg of total RNA in a 20 μL reaction, and diluted 4-fold with water.

Quantitative real-time RT-PCR was performed using SYBR premix EX Taq (Takara, Japan). *ACT1* was used as an endogenous control gene to normalize expression of the other genes. Primers used in real-time RT-PCR were shown in supplementary Table 1. The PCR program was as follows: 30 s at 95 °C, followed by 40 cycles of 10 s at 95 °C, 30 s at 60 °C.

### Transmission electron microscopy study

After 14 days of treatment, leaf segments without veins and root tips (8–10 each per treatment) were collected from randomly selected seedlings and then fixed overnight in 2.5% glutaraldehyde (v/v) in 0.1 M PBS (sodium phosphate buffer, pH 7.4) and washed three times with the same PBS. Then the samples were post fixed in 1% OsO_4_ [osmium (VIII) oxide] for 1 hr. and washed three times in 0.1 M PBS (pH 7.4), with 10-min intervals between each washing. Then, with 15–20 min intervals, the samples were dehydrated in a graded series of ethanol (50%, 60%, 70%, 80%, 90%, 95% and 100%) and at the end washed by absolute acetone for 20 min. The samples were then infiltrated and embedded in Spurr’s resin overnight. After heating at 70 °C for 9 hr, ultrathin sections (80 nm) of specimens were prepared and mounted on copper grids for viewing by a transmission electron microscope (JEOLTEM- 1230EX) at an accelerating voltage of 60.0 kV.

### Statistical analysis

Treatments were arranged in factorial experimental in completely randomize design, All values described in results section were mean of three replications ± standard deviation (SD). Percentage data were arcsin-transformed before analysis according to ŷ = arcsin [sqr (x/100)]. The data were analyzed using SPSS v16.0 (SPSS, Inc., Chicago, IL, USA). Analysis of variance (ANOVA) was carried out, followed by Duncan’s multiple range test between the means of treatments to determine the significant difference at the *p* < *0.05* and 0.01 level between mean values.

## Additional Information

**How to cite this article**: Salah, S. M. *et al.* Seed priming with polyethylene glycol regulating the physiological and molecular mechanism in rice (*Oryza sativa* L.) under nano-ZnO stress. *Sci. Rep.* **5**, 14278; doi: 10.1038/srep14278 (2015).

## Supplementary Material

Supplementary Information

## Figures and Tables

**Table 1:** Effect of seed priming with PEG (30%) on germination percentage (GP%), germination index (GI), Energy of germination (EG%), mean germination time (MGT d.) and leaf surface area (cm^2^) of two rice cultivars under different ZnO nanorods concentrations.

Cultivars	Priming withPEG (%)	nano-ZnO conc.(mg L^−1^)	GP (%)	GI	EG (%)	MGT (days)	Leaf surface area(cm^2^)
Zhu Liang You 06	0	0	90.00 ± 2.00 e	96.66 ± 2.30 a–c	81.33 ± 2.30 g	6.13 ± 0.05 f	3.30 ± 0.11 i
		250	86.00 ± 3.46 f	95.00 ± 2.00 bc	76.00 ± 4.00 h	7.19 ± 0.08 c	2.65 ± 0.39 j
		500	82.00 ± 3.46 g	90.33 ± 4.50 d	76.00 ± 0.05 h	7.71 ± 0.10 b	2.67 ± 0.04 j
		750	52.00 ± 2.00 j	57.33 ± 3.21 g	72.00 ± 0.09 i	8.53 ± 0.32 a	2.45 ± 0.13 j
	30	0	95.33 ± 1.15 b–d	98.33 ± 0.57 ab	100.00 ± 0.04 a	4.53 ± 0.25 j	4.95 ± 0.05 d
		250	93.33 ± 1.15 c–e	97.00 ± 0.08 a–c	94.66 ± 2.30 cd	5.50 ± 0.10 h	4.60 ± 0.10 e
		500	92.00 ± 0.05 de	94.33 ± 2.30 bc	92.00 ± 0.08 de	6.30 ± 0.10 ef	4.23 ± 0.20 fg
		750	66.00 ± 2.00 h	68.66 ± 3.05 f	88.00 ± 0.07 f	6.88 ± 0.07 d	4.03 ± 0.05 gh
Qian You No. 1	0	0	96.00 ± 0.05 bc	96.00 ± 0.03 a–c	96.00 ± 4.00 bc	5.43 ± 0.14 h	4.41 ± 0.10 ef
		250	92.00 ± 0.03 de	95.00 ± 0.08 bc	96.00 ± 0.08 bc	5.80 ± 0.10 g	4.13 ± 0.15 gh
		500	90.00 ± 0.05 e	93.00 ± 0.07 cd	90.66 ± 2.30 ef	6.38 ± 0.07 e	3.88 ± 0.07 h
		750	58.00 ± 2.08 i	60.00 ± 2.00 g	88.00 ± 0.07 f	6.88 ± 0.07 d	3.38 ± 0.02 i
	30	0	100.00 ± 0.06 g	100.00 ± 0.07 a	100.00 ± 0.09 a	4.16 ± 0.03 k	5.90 ± 0.10 a
		250	98.00 ± 0.05 ab	98.00 ± 0.09 ab	100.00 ± 0.09 a	4.71 ± 0.10 j	5.61 ± 0.17 b
		500	98.00 ± 0.07 ab	98.00 ± 0.07 ab	99.00 ± 0.05 ab	5.14 ± 0.05 i	5.26 ± 0.05 c
		750	84.00 ± 4.00 fg	84.00 ± 4.00 e	98.00 ± 0.07 ab	5.40 ± 0.10 h	5.03 ± 0.05 cd

The same letters within a column indicate there was no significant difference at a 95% probability level at the *p *<* 0.05* level, respectively.

**Table 2:** Effect of seed priming with PEG (30%) on root and shoot length (cm), seedling fresh and dry weight (g) and seedling vigor index of two rice cultivars under different ZnO nanorods concentrations.

Cultivars	Priming withPEG (%)	nano-ZnO conc.(mg L^−1^)	Root length(cm)	Shoot length(cm)	SFW (g)	SDW (g)	SVI
Zhu Liang You 06	0	0	9.16 ± 0.61 f–h	7.33 ± 0.15 de	0.073 ± 0.01 fg	0.043 ± 0.02 b	1484 ± 29.6 f
		250	8.60 ± 0.36 h–j	6.60 ± 0.36 ef	0.070 ± 0.01 gh	0.042 ± 0.05 b	1306 ± 69.9 g
		500	8.03 ± 0.25 j	6.03 ± 0.25 f	0.066 ± 0.01 i	0.042 ± 0.05 b	1154 ± 88.6 h
		750	5.03 ± 0.45 l	3.83 ± 1.44 g	0.052 ± 0.02 k	0.041 ± 0.03 b	425 ± 21.5 j
	30	0	10.93 ± 0.11 ab	8.93 ± 0.11 ab	0.087 ± 0.05 b	0.043 ± 0.01 b	1893 ± 22.8 bc
		250	10.53 ± 0.25 bc	7.86 ± 0.92 cd	0.083 ± 0.01 cd	0.042 ± 0.01 b	1717 ± 66.2 de
		500	9.80 ± 0.20 d–f	7.83 ± 0.28 cd	0.080 ± 0.01 d	0.042 ± 0.05 b	1625 ± 53.1 e
		750	9.16 ± 0.28 f–h	7.26 ± 0.46 de	0.073 ± 0.02 fg	0.041 ± 0.01 b	1084 ± 27.8 h
Qian You No. 1	0	0	9.30 ± 0.62 e–g	7.70 ± 0.17 d	0.076 ± 0.01 ef	0.046 ± 0.05 a	1631 ± 76.1 e
		250	8.73 ± 0.32 g–i	6.73 ± 0.51 ef	0.073 ± 0.01 fg	0.046 ± 0.02 a	1422 ± 76.4 fg
		500	8.23 ± 0.20 ij	6.23 ± 0.20 f	0.069 ± 0.01 h	0.043 ± 0.05 b	1302 ± 37.4 g
		750	5.76 ± 0.68 k	3.76 ± 0.68 g	0.056 ± 0.02 j	0.041 ± 0.01 b	572 ± 36.6 i
	30	0	11.26 ± 0.25 a	9.26 ± 0.25 a	0.090 ± 0.05 a	0.046 ± 0.02 b	2053 ± 50.3 a
		250	10.73 ± 0.25 ab	8.73 ± 0.25 a–c	0.086 ± 0.01 bc	0.046 ± 0.01 b	1907 ± 49.3 b
		500	10.06 ± 0.32 cd	8.06 ± 0.32 b–d	0.083 ± 0.01 cd	0.044 ± 0.01 b	1776 ± 62.7 cd
		750	9.90 ± 0.17 c–e	7.90 ± 0.17 cd	0.076 ± 0.03 e	0.042 ± 0.05 b	1408 ± 193.9 fg

The same letters within a column indicate there was no significant difference at a 95% probability level at the *p *<* 0.05* level, respectively.
