# Supplementary material for: Seed priming with polyethylene glycol regulating the physiological and molecular mechanism in rice (Oryza sativa L.) under nano-ZnO stress
Source: Sci Rep. 2015 Sep 30;5:14278. doi: 10.1038/srep14278 (PMC4588511; doi:10.1038/srep14278)
Supplement: Supplementary Information [file srep14278-s1.pdf]

Seed priming with polyethylene glycol regulating the physiological and molecular mechanism in rice (*Oryza sativa* L.) under nano-ZnO stress

Sheteiwy Mohamed Salah[1,2], Guan Yajing[1], Cao Dongdong[3], Li Jie[1], Nawaz Aamir[1,4], Hu Qijuan[1], Hu Weimin[1], Ning Mingyu[5], Hu Jin[1,*]

[1]Seed Science Center, College of Agriculture and Biotechnology, Zhejiang University, Hangzhou 310058, China, [2]Department of Agronomy, Faculty of Agriculture, Mansoura University, Mansoura 35516, Egypt, [3]Zhejiang Nongke Seed industry Limited Company, Hangzhou, 310021, China, [4]Faculty of Agricultural Sciences and Technology, Bahauddin Zakariya University Multan, 60000 Pakistan; [5]National Agricultural Technology Extension Service Center, China.

[*] Corresponding author. Tel.: +86 57188982318; fax: +86 57188982318; E-mail address: jhu@zju.edu.cn

Table S1

Sequences of oligonucleotide primers used in QRT-PCR

Locus	Primer name	Sequence (5′-3′)
Os03g0285700	Os-APXa-F	GCTGAGTGACCCTGCCTTCC
	Os-APXa-R	CCCCAGTTCGGAGAGCTTGA
Os07g0694700	Os-APXb-F	ACCCTGGGAAGATGCCACAA
	Os-APXb-R	GCCTTCCTTCTCGCCACTCA
Os02g0115700	Os-CATa-F	GGAAGCTGTTCGTCCAGGTGAT
	Os-CATa-R	TCCGGCCATGTCTTGGTGTC
Os06g0727200	Os-CATb-F	TTCTCCAGCGGTGGGTTGAT
	Os-CATb-R	CCAAGGGACGCATCACACTG
Os03g0131200	Os-CATc-F	CACTCTCTGACCCTCGCCTCA
	Os-CATc-R	AGACGGCTCGCCAGTTTCTG
Os03g0351500	Os-SOD1-F	GGGAGATGGTCCCACCACTG
	Os-SOD1-R	GGCCCAGTTGACATGCAACC
Os03g0219200	Os-SOD2-F	CAACTCTACCGGGCCCCATT
	Os-SOD2-R	GGCTACTATGTTTCCCAGGTC
Os07g0665200	Os-SOD3-F	GCCAGATCCCCCTTACTGGA
	Os-SOD3-R	CCCCAGCATTTCCAGTGGTC
Os03g0718100	Os-ACT1-F	CTTCATAGGAATGGAAGCTGCGGGT
	Os-ACT1-R	CGACCACCTTGATCTTCATGCTGCT

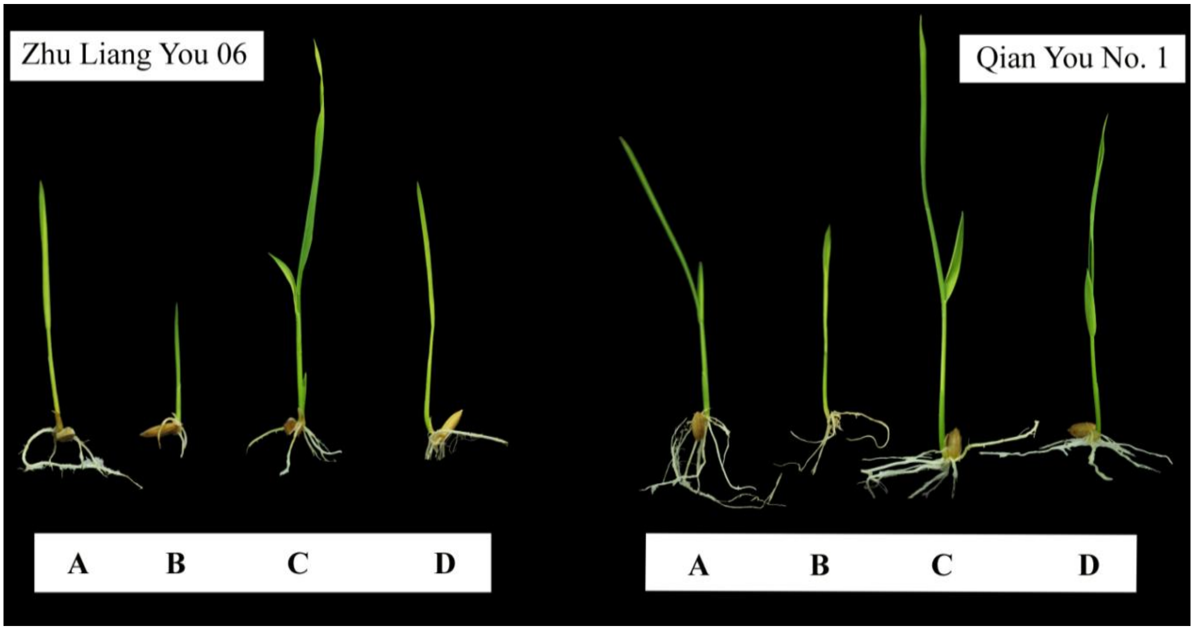

Fig. S1. Seedlings phenotypes of *Oryza sativa* (Zhu Liang You 06 and Qian You No.1)two weeks old, exposed to different concentrations of nano-ZnO.A [without priming and without stress (control)], B [without priming and high stress (750 mg L^{-1})], C [priming with PEG and without stress] and D [priming with PEG and high stress (750 mg L^{-1})].
